# Antigen-Specific Antibody Glycosylation Is Regulated via Vaccination

**DOI:** 10.1371/journal.ppat.1005456

**Published:** 2016-03-16

**Authors:** Alison E. Mahan, Madeleine F. Jennewein, Todd Suscovich, Kendall Dionne, Jacquelynne Tedesco, Amy W. Chung, Hendrik Streeck, Maria Pau, Hanneke Schuitemaker, Don Francis, Patricia Fast, Dagna Laufer, Bruce D. Walker, Lindsey Baden, Dan H. Barouch, Galit Alter

**Affiliations:** 1 Ragon Institute of MGH, MIT and Harvard, Cambridge, Massachusetts, United States of America; 2 Military HIV Research Program, Walter Reed Medical Research Institute, Bethesda, Maryland, United States of America; 3 Crucell, Leiden, Netherlands; 4 Academic Medical Center of the University of Amsterdam, Amsterdam, The Netherlands; 5 Global Solutions for Infectious Diseases, South San Francisco, California, United States of America; 6 International AIDS Vaccine Initiative, New York, New York, United States of America; 7 Department of Medicine, Brigham and Women’s Hospital, Boston, Massachusetts, United States of America; 8 Center for Virology and Vaccine Research, Beth Israel Deaconess Medical Center, Boston, Massachusetts, United States of America; University of Zurich, SWITZERLAND

## Abstract

Antibody effector functions, such as antibody-dependent cellular cytotoxicity, complement deposition, and antibody-dependent phagocytosis, play a critical role in immunity against multiple pathogens, particularly in the absence of neutralizing activity. Two modifications to the IgG constant domain (Fc domain) regulate antibody functionality: changes in antibody subclass and changes in a single N-linked glycan located in the CH2 domain of the IgG Fc. Together, these modifications provide a specific set of instructions to the innate immune system to direct the elimination of antibody-bound antigens. While it is clear that subclass selection is actively regulated during the course of natural infection, it is unclear whether antibody glycosylation can be tuned, in a signal-specific or pathogen-specific manner. Here, we show that antibody glycosylation is determined in an antigen- and pathogen-specific manner during HIV infection. Moreover, while dramatic differences exist in bulk IgG glycosylation among individuals in distinct geographical locations, immunization is able to overcome these differences and elicit antigen-specific antibodies with similar antibody glycosylation patterns. Additionally, distinct vaccine regimens induced different antigen-specific IgG glycosylation profiles, suggesting that antibody glycosylation is not only programmable but can be manipulated via the delivery of distinct inflammatory signals during B cell priming. These data strongly suggest that the immune system naturally drives antibody glycosylation in an antigen-specific manner and highlights a promising means by which next-generation therapeutics and vaccines can harness the antiviral activity of the innate immune system via directed alterations in antibody glycosylation in vivo.

## Author Summary

Accumulating evidence points to a critical role for non-neutralizing antibody functions in protective immunity against a variety of pathogens, including HIV. Non-neutralizing antibody function is controlled by antibody constant domain interactions with Fc receptors, which itself is regulated via changes in antibody subclass/isotype selection or antibody glycosylation. This study specifically aimed to determine whether glycosylation of IgG is naturally tuned to target distinct pathogens or antigens and whether this activity can be actively modulated to direct antibody effector function. The study clearly demonstrates that the immune system naturally exploits unique IgG glycosylation profiles to target distinct pathogens and antigens and that this activity can be actively manipulated via vaccination. Moreover, because different vaccines drive unique glycosylation profiles, future studies that define the specific signals that control antibody glycosylation may lead to the generation of next-generation therapeutic interventions that can leverage and specifically direct the killing activity of the innate immune system, targeting HIV and beyond.

## Introduction

Mounting evidence points to a critical role for non-neutralizing antibody effector function, such as antibody-dependent cellular cytotoxicity (ADCC), antibody-dependent cellular phagocytosis (ADCP) and complement-dependent cytotoxicity (CDC), in protection against [1], and control of HIV [2], influenza [3], Ebola virus [4], and bacterial infections [5]. Earlier work suggests that potent, long-lived antibody effector activity is driven by IgG1 antibodies [6], the dominant subclass in the blood [7]. However, as all vaccinated and infected individuals ultimately produce IgG1 antibodies, it is unclear why some IgG1 responses provide protective immunity while others provide limited immunity at the same titers. While emerging data suggest that the co-selection of additional antibody subclasses, such as the most functional subclass, IgG3, may collaborate to direct more effective immune complex–based activity [8], IgG3 is cleared rapidly from the systemic circulation [9], arguing that sustained levels of some, but not other IgG1 antibodies may represent the critical determinant of protective immunity against HIV. Thus, defining how the immune system naturally tunes IgG1 represents a critical step for the development of more effective strategies to harness the immune system to prevent or control HIV infection.

Every IgG antibody is glycosylated at a single asparagine residue within the CH2 domain of the constant region (in the crystallizable fragment, Fc), and data from the monoclonal therapeutic community suggest that these changes potently alter the inflammatory profile and effector functions of the antibody [10]. The antibody glycan consists of variable levels of four sugar subunits (galactose, sialic acid, fucose and an N-acetylglucosamine that bisects the arms of the structure (b-GlcNAc)), each of which alters the affinity of the antibody for innate immune receptors, including Fc receptors found on all innate immune cells [11]. For example, changes in fucose and the b-GlcNAc play a critical role in modulating monoclonal therapeutic antibody effector function, where a lack of fucose [12], the addition of the b-GlcNAc [13], and elevated sialic acid [14] increases ADCC activity. In contrast, agalactosylated polyclonal antibodies are associated with increased inflammation in HIV [15,16] and chronic autoimmune conditions [17] and agalactosylated monoclonal therapeutics are known to drive enhanced complement binding and activation [18]. Conversely, the presence of higher levels of galactose provides the scaffold for the addition of terminal sialic acid groups, that are thought to drive anti-inflammatory activity through binding to lectin-like receptors [19], though there is some controversy in the field as to whether IVIG’s anti-inflammatory effect is due to sialylation alone [20–22].

Thus, while the antibody therapeutics field has clearly demonstrated that alterations in antibody glycosylation is a critical mechanism for improving therapeutic efficacy via the augmentation of effector function [6,13,23] or through the alteration of inflammation in rheumatoid arthritis treatment [19], it is still unclear whether antibody glycosylation is actively regulated in vivo. While, recent studies on antigen specific antibodies have shown that antigen-specific antibodies are induced with distinct antibody glycan profiles, it is still unclear whether distinct antibodies within the same individual are programmed with unique glycosylation profiles aimed at enhancing particular effector functions. However, given the emerging data pointing to distinct antibody glycan profiles on antigen-specific antibodies compared to bulk circulating antibodies [15,24], it is possible that antibody glycosylation may be actively controlled by the immune system. Moreover, over 30 different glycan structures have been identified in naturally produced antibodies, each with the theoretical capacity to drive distinct effector functional profiles [25,26], that may be selected immunologically in disparate manners to drive unique effector functions. Thus this study aimed to determine whether antibody glycosylation is differentially tuned against specific pathogens and/or antigenic targets and whether antibody glycosylation could be actively directed through immunological priming. We demonstrate different glycoprofiles on particular antigen- and pathogen-specific antibodies, clearly illustrating unique antibody glycan profiles against individual antigens, each of which was distinct from that present in bulk circulating antibodies, linked to distinct antibody effector functions, pointing to antigen-specific regulation of antibody glycosylation. Furthermore, while bulk circulating antibody Fc glycosylation was dramatically different in geographically distinct populations, immunization with a viral vector-based vaccine induced remarkably similar antigen-specific antibody glycan profiles on vaccine-specific antibodies induced at all three geographic sites. Conversely, distinct antigen-specific IgG glycosylation profiles were induced by a protein–based HIV vaccine, suggesting that antibody glycosylation is selectively tuned at the time of vaccination by distinct inflammatory signals co-delivered at the time of B cell priming. Collectively, these data argue that IgG glycosylation is elicited differently by specific pathogens, antigens, and immune signals to selectively and specifically induce the targeted antibody functional profiles. Thus the regulation of antibody glycosylation represents a potentially novel means by which next-generation therapeutic or vaccine strategies may selectively direct the immune regulatory and killing activity of antibodies.

## Results

### IgG Fc glycosylation occurs in an antigen- and pathogen- specific manner

**Fig 1:**
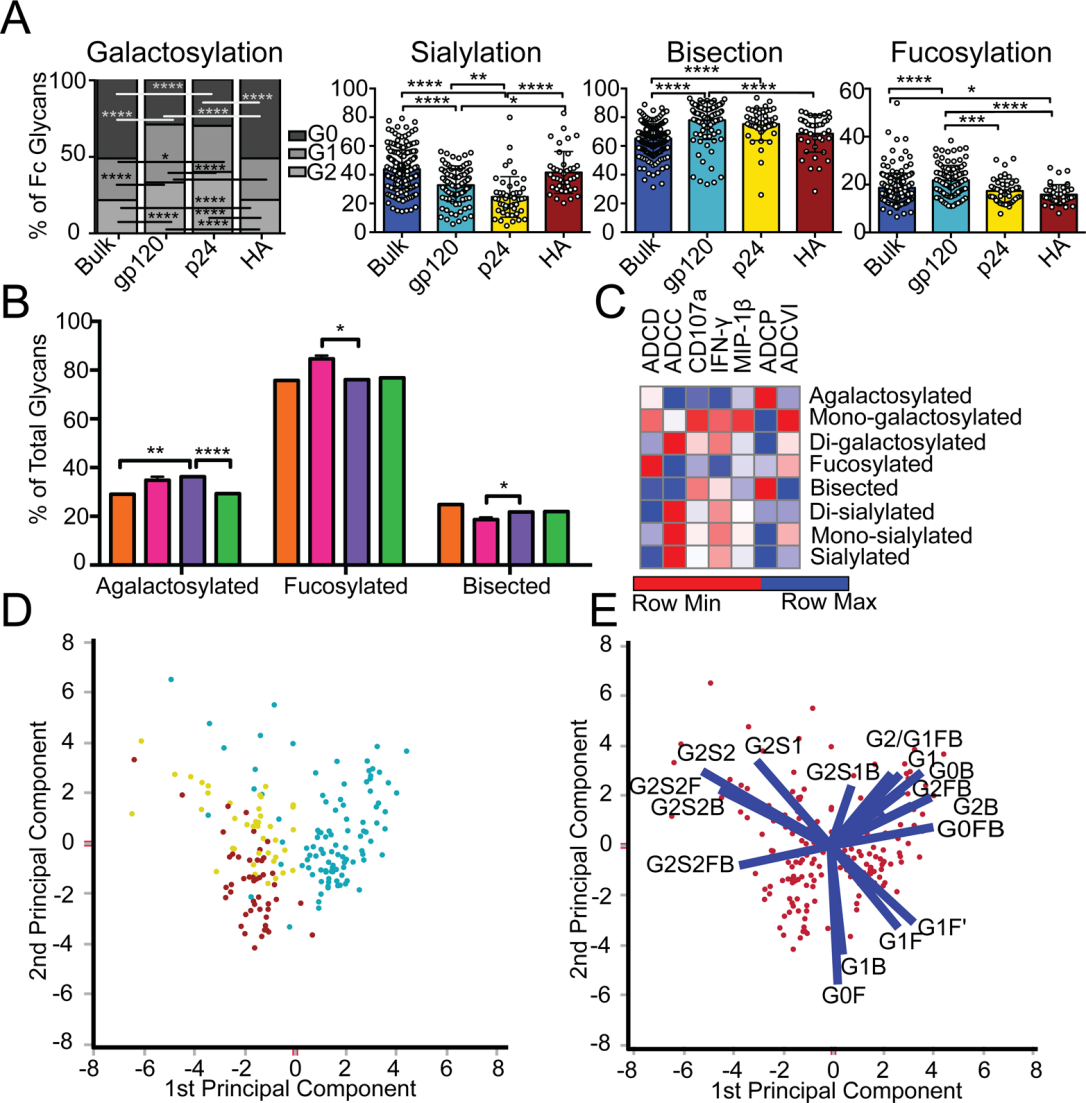
Antibody glycosylation is programmed in an antigen-specific manner. (A) Glycosylation was assessed on whole bulk-circulating and antigen-specific IgG antibodies directed against gp120 (n = 103), p24 (n = 47), and HA (n = 40) isolated from a cohort of 193 HIV-infected subjects. The dot plots represent the percent of glycan structures that contain galactose (G0 = agalactosylated, G1 = mono-galactosylated, G2 = di-galactosylated) or that contain sialic acid (S = sialylated), fucose (F = fucosylated), or a bisecting GlcNAc (B = bisected). Differences between groups were compared using Kruskal-Wallis test with Dunn’s multiple comparison’s test (*p<0.05, **p<0.01, ***p<0.001, ****p<0.0001). (B) Differences in glycosylation for gp120-specific antibodies from chronic treated (orange), chronic untreated (pink), viremic controller (purple) and elite controller (green) HIV patients were compared using two-way ANOVA with Tukey’s multiple comparison test (*p<0.05, **p<0.01, ***p<0.001, ****p<0.0001). (C) Antibody glycosylation levels on gp120-specific antibodies among chronic HIV-infected individuals were measured along with seven different gp120-specific effector functions. The correlations between gp120-specific antibody glycosylation and gp120-specific functional parameters were assessed using spearman correlations. Antibody dependent complement deposition (ADCD), antibody dependent cellular cytotoxicity (ADCC), antibody mediated NK cell activation (degranulation-CD107a, IFN-γ, or MIP-β secretion), antibody dependent cellular phagocytosis (ADCP), and antibody dependent cellular viral inhibition (ADCVI) are depicted. Significant correlations were identified for: Fucose—ADCC*, Di-sialylated—ADCC**, mono-galactosylated—ADCP*, Di-galactosylated—ADCP**, Bisection—ADCP* and Mono-sialylated—ADCP** (*p<0.05, **p<0.01, ***p<0.001, ****p<0.0001). (D), (E) To gain a multivariate sense of the overall glycan profile differences among antigen-specific antibodies, antigen-specific antibody glycan profiles (gp120- (teal), HA- (yellow) and p24- (red) specific antibodies) were compared using principle components analysis (PCA). Each dot on the score plot (D) represents an antigen-specific antibody glycan profile from a single individual and the loadings plot (E) shows the contribution of individual analyzed glycan structures to driving the separation between the antigen-specific antibody glycan profiles. Vector length represents the magnitude of individual glycan structure effects on overall separation in antibody glycan profiles, with longer vectors represent features that are further from the mean and that drive a larger effect on separating antibody glycan profiles. Moreover, location on the loadings plot is identical to location on the score plot, determined by the collective influence of all vectors. This analysis accounts for 42.9% of the glycosylation variation across the antigen-specific antibody specificities.

Inflammatory diseases [27] and viral infections [10,11] drive an overall shift in bulk circulating antibody glycosylation. Likewise, recent studies have highlighted that unique glycan profiles emerge on antigen-specific antibodies [15,24]. However, whether all antigen-specific antibodies exhibit the same glycan profiles, tuned exclusively by inflammation, or whether antigen-specific antibody populations are tuned in an antigen- and/or pathogen- specific manner is unclear. Thus, HIV envelope (gp120)-, HIV capsid (p24)-, and influenza envelope (HA)-specific antibodies were selectively enriched from a population of 193 HIV-infected patients, and glycan profiling was performed on enzymatically removed glycans by capillary electrophoresis (S1 Fig, S1 Table). Remarkably, antibody glycosylation differed not only between antigen-specific antibodies and bulk circulating antibodies, but also among the three different antigen-specific antibody populations (Fig 1A). Specifically, as previously reported, gp120-specific antibodies possessed elevated levels of agalactosylated glycans and slightly increased fucosylated and bisected glycans with a decrease in sialylated structures compared to bulk antibodies (Fig 1A). Collectively, this combination of sugars points to the induction of a more functional (elevated b-GlcNAc) and more inflammatory (low galactose and sialic acid) glycan on gp120-specific antibodies [15,16]. Thus, based on known glycan structure:function relationships [13,28], gp120-specific antibodies exhibit a slightly inflammatory asialylated glycan poised to direct ADCC and complement-mediated killing via elevated b-GlcNAc levels.

However, p24-specific antibodies exhibited an even more exaggerated inflammatory profile than gp120-specific antibodies (Fig 1A). p24-specific antibody glycans included significantly higher levels of agalactosylated glycans and slightly more fucosylation, as compared to bulk antibody glycans (Fig 1A). These antibodies exhibited low levels of sialic acid and comparable levels of b-GlcNAc to the bulk circulating antibodies. Thus, p24-specific antibodies are selectively tuned to express a highly inflammatory agalactosylated glycan. In contrast to gp120- and p24-specific antibodies, influenza-specific antibodies exhibited a significantly different glycan profile, marked by significantly increased galactosylation (Fig 1A) and sialylation and reduced b-GlcNAc. Thus, influenza-specific antibodies exhibited a third glycan profile, that like IVIG, may be tipped towards an anti-inflammatory glycan [10,19] that may be deliberately tuned to drive enhanced ADCC via reduced fucose [28]. Interestingly, no correlation was observed among glycan patterns selected on gp120- or HA-specific antibodies (S2 Fig) arguing that antigen-specific antibody glycosylation is selected independently in each individual and is not influenced by the host’s genetic or pre-infection background. Thus, based on univariate analyses, comparing the incorporation of individual sugars into the antibody glycan, antibody glycosylation varies significantly among antigen-specificities in HIV infected persons.

Beyond differences at the antigen-specific level, differences were previously observed in gp120-specific antibody glycosylation profiles among a small group of HIV infected patients with differential clinical progression profiles [15]. Similar differences in antibody glycan-profiles were observed within this larger patient population (Fig 1B), with elevated agalactosylation among all spontaneous controllers and elevated bisection among the chronic treated patients. However, interestingly, no between HIV group differences were observed among the influenza-specific antibody glycan profiles (S3 Fig), demonstrating disease specific nature of antibody glycan tuning.

While changes in antibody glycosylation and their effect on antibody effector function has been clearly illustrated in the context of monoclonal therapeutics [6], less is known about the impact of glycan-structure changes, which are often small, in polyclonal antibody populations. Thus we next sought to define whether the observed glycan-profile differences impacted antibody effector function. Seven gp120-specific antibody effector functions, including: antibody dependent complement deposition (ADCD), ADCC, NK degranulation associated CD107a surface expression, interferon-γ (IFN-γ) and macrophage inflammatory protein 1β (MIP-1β) release, ADCP and antibody dependent cellular viral inhibition (ADCVI) were assessed in a group of chronically untreated HIV infected patients for whom sufficient amounts of plasma were available for functional profiling. Thus gp120-specific antibody glyco-profiles and gp120-specific antibody functionality were assessed in parallel to determine the relationship between glycosylation and antibody functionality against the same antigenic target in polyclonal pools of antibodies. The glycan profile:function correlational analyses showed a number of relationships (Fig 1C). Interestingly, known relationships previously demonstrated for monoclonal therapeutics [12,13,29], such as the association between: 1) low fucose and high ADCC, high bisecting GlcNAc and high ADCC, and 3) low galactose and complement activation, were observed in polyclonal gp120-specific antibodies (Fig 1C). Additionally, novel correlations were also observed including a significant positive association between di-sialylation and ADCC, agalactosylation and bisection were positively correlated with phagocytic activity, and mono-sialylation and di-galactosylation were associated with antibody mediated viral inhibition (Fig 1C). These data suggest that polyclonal antibody glycan structure shifts clearly result in alterations in antibody effector function that may be actively regulated during an immune response to direct enhanced clearance and control in an antigen-specific manner.

To gain a more complete understanding of the differences in glycosylation between different antigen-specific antibody populations, we used principle component analysis (PCA) to generate integrated multivariate glycan profiles for each antibody population. PCA linearly transforms multi-dimensional measurements into linear coordinates, called principle components. We can plot the first two principle components, which account for the greatest variation, onto a two-dimensional plot to define multivariate differences among groups (overlapping dots reflect similar profiles whereas non-overlapping dots represent different overall glycan profiles). Specifically, PCA demonstrated that the three antigen specificities separated as distinct antigen-specific antibody glycan profiles (Fig 1D), with limited overlap, suggesting that each antigen-specific antibody population is induced with a unique glycan profile. Moreover, the vectors on the loadings plot (Fig 1E), illustrate the strength of the contribution of each glycan structure in driving the separation in the overall glycan profiles, highlighting the unique nature of selective enrichment of di-sialylated glycans among HA-specific antibodies, elevated G0F/G1B glycans among the p24-specific antibodies, and G1/G0FB glycans in the gp120-specific antibody population.

Overall, these data strongly argue that antibody glycosylation is calibrated at an antigen-specific level within the same individual and that that each profile is distinct from one another and from bulk circulating antibodies (Fig 1). These data therefore suggest that antibody glycosylation may be tuned actively during the induction of an immune response, to generate an antigen/pathogen appropriate effector response. However whether antibody glycosylation can be actively manipulated is still unclear.

### Bulk IgG Fc-glycosylation varies geographically

To determine whether antibody glycosylation is programmable in a reproducible manner we turned to a vaccine trial using the experimental Ad26/Ad35 expressing an HIV Envelop protein A (the B003/IPCAVD-004/HVTN091 trial) that was conducted at sites in the United States, Kenya/Rwanda (East Africa), and South Africa. As IgG bulk and antigen-specific glycosylation differ dramatically in inflammatory diseases [27] and infectious diseases [15], bulk circulating antibody Fc glycosylation profiles were initially compared across the sites to ascertain baseline differences between the vaccine populations.

**Fig 2:**
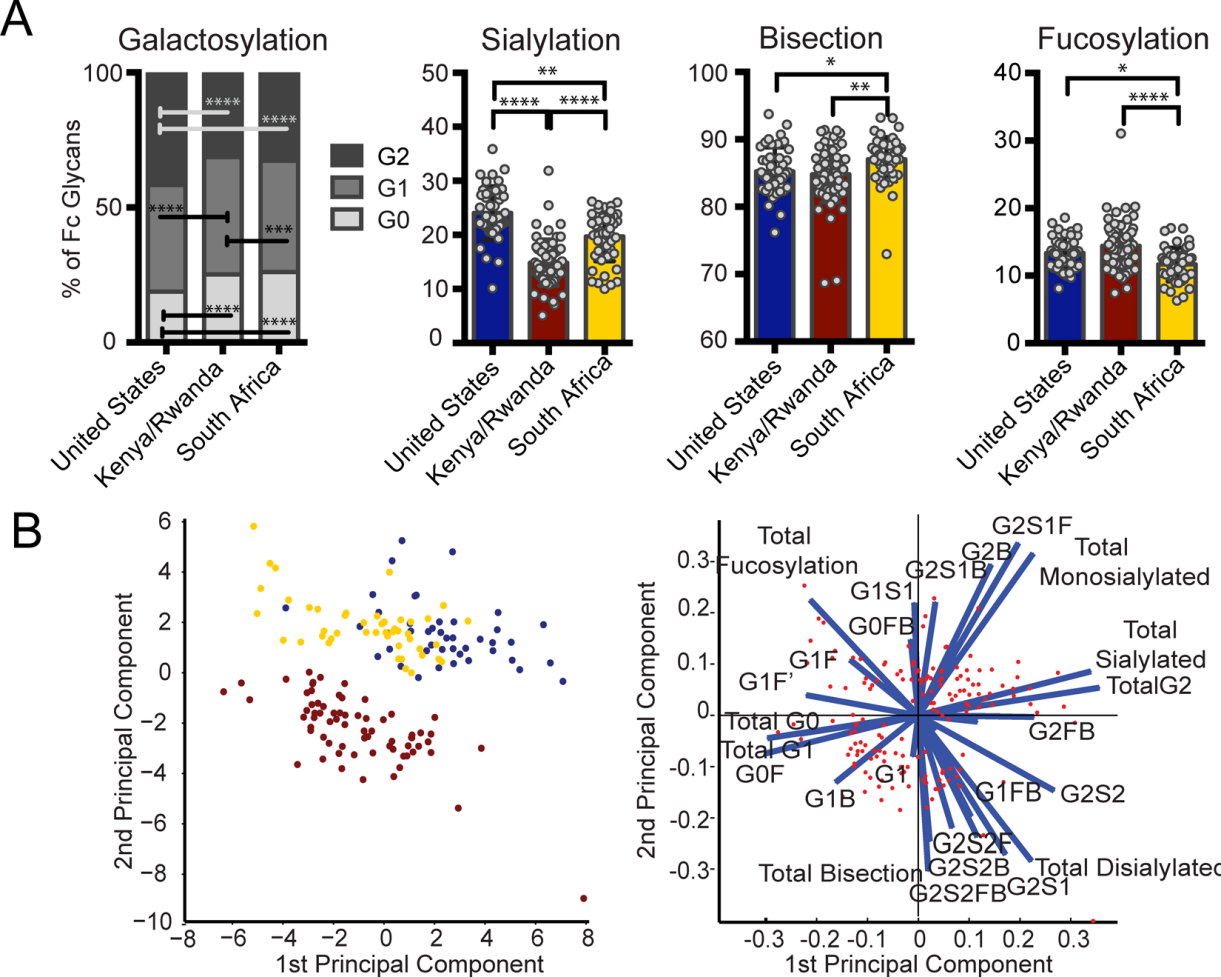
Geographic location affects bulk IgG glycosylation. Bulk IgG glycosylation was assessed in subjects from three regions: Unite States (blue, n = 43), Kenya and Rwanda (maroon, n = 69), and South Africa (yellow, n = 47). (A) Bulk antibody Fc glycosylation in vaccine recipients from each of the three regions was measured via capillary electrophoresis, and the mean proportion of total galactosylated, sialylated, fucosylated, and bisected structures was compared using Kruskal-Wallis one-way ANOVA (*p<0.05, **p<0.01, ***p<0.001, ****p<0.0001). (B) Multivariate comparison of antibody Fc glycosylation among the three geographic sites was performed using PCA. The score plot (left panel) depicts the principal component analysis of samples collected in the three regions (each dot represents a vaccinee, and colors are as described above), and the loadings plot of the PCA (right panel) shows the contribution of particular glycan structures to driving the observed separation, where longer arrows signify a greater contribution to separating glycan profiles. This PCA describes 55% of the total variance among these samples.

Significant differences in bulk IgG Fc galactosylation and sialylation were observed among individuals in the three regions (Fig 2A). In particular, individuals from both African regions exhibited significantly higher proportions of agalactosylated (G0) Fcs. While both African groups displayed lower sialylation than US vaccinees, East Africans had the lowest bulk antibody sialylation. Given the role of low galactose and sialylation in determining the inflammatory activity of antibodies [10], these data suggest that bulk antibody glycosylation in Africans is associated with inflammatory glycosylation, with East Africans having the most inflammatory profile. Since the bulk IgG population is made up of a large array of antigen-specific antibodies corresponding to the pathogens encountered by an individual, these differences may correspond to differences in genetic background, diet, and/or exposure to pathogens at each geographical location.

As mentioned above, changes in fucose and b-GlcNAc alter antibody function [12,13]. Interestingly, in the South African cohort, we observed higher fucosylation of bulk Fc compared to either of the two other groups (Fig 2A). Additionally, South Africans had lower b-GlcNAc compared to both groups. Given the low functional activity of antibodies with high fucose and low b-GlcNAc containing glycans, these results highlight that even within a single continent, significant differences may arise in antibody glycosylation of bulk antibodies, not only in sugars that modulate inflammation, but also among sugars that are critical for driving antibody functionality.

Principle component analysis demonstrated largely non-overlapping antibody glycosylation profiles among vaccinees at each of the three sites in their bulk circulating antibody glycosylation profiles (Fig 2B), demonstrating that fundamentally different glycosylation profiles exist within each geographic region. The separation was largely driven by inflammatory glycan structures as shown by the length of the vectors on the loadings plot. For example, G0 structures, associated with inflammation [17], were largely associated with Kenya/Rwanda, whereas G2 structures, that are thought to be less inflammatory, were enriched among vaccinees from the US. Interestingly, the East African vaccinees separated completely from the other groups, while the South African and American recipients overlapped slightly in their bulk Fc-glycan profiles, suggesting that while variation in bulk Fc glycosylation exists among all groups, there are greater differences between East Africans and the other two populations, which are potentially related to a multitude of variables including distinct genetics [25], endemic infections [30], differences in diet [31], and other environmental factors. Overall, these data strongly suggest that baseline inflammation may alter the bulk antibody glycan profiles, potentially pre-determining the glycan profile of newly elicited antibodies.

### Glycosylation on vaccine-specific antibodies is distinct from bulk antibody glycosylation

**Fig 3:**
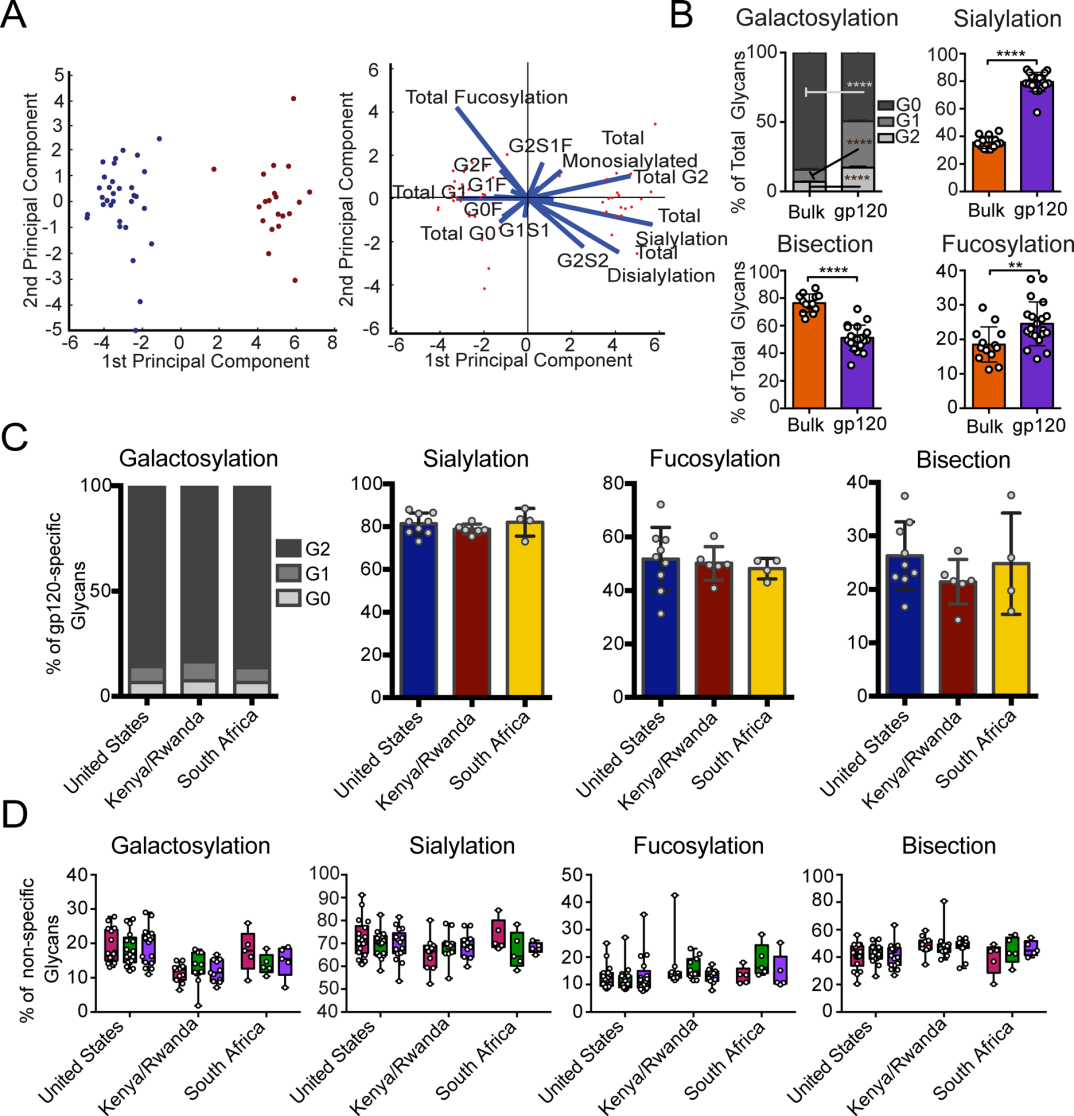
Vaccine-elicited antibody glycosylation profiles are distinct from bulk antibody glycosylation. Viral vector–induced gp120-specific and influenza specific antibodies were isolated from vaccinees, and the attached glycans were analyzed by capillary electrophoresis. (A) Multivariate PCA was used to compare bulk antibody glycoprofiles (blue, n = 32) and vaccine-elicited antigen-specific antibody glycoprofiles (maroon, n = 20), and both the scores plot (left) and loadings plot (right) are shown. This analysis describes 69% of the variation. (B) The mean proportions of bulk and vaccine-elicited antibody glycan were compared using students two-tailed paired t tests (n = 13 for bulk, n = 20 for gp120 (*p<0.05, **p<0.01, ***p<0.001, ****p<0.0001) (C) The mean proportions of vaccine-elicited antibody glycan structures were compared across vaccine groups using Kruskal-Wallis ANOVA (n = 9 for United States, n = 6 for Kenya/Rwanda, n = 4 for South Africa). No statistically significant differences were found. (D) The mean proportions of influenza-specific antibody glycans at baseline (magenta), post-first (green), and post-boost (purple) vaccine timepoints were compared using non-parametric two-way ANOVA (n = 18 for United States, n = 11 for Kenya/Rwanda, n = 5 for South Africa). No significant differences were found between the three timepoints for either antigen or glycan type.

We next aimed to determine whether antigen-specific antibodies induced via vaccination exhibited distinct glycosylation profiles to bulk circulating antibodies, in a manner analogous to the distinct antigen-specific antibodies seen in HIV-infected subjects (Fig 1) Thus, the viral vector–induced antigen-specific antibodies were enriched from bulk IgG using vaccine-matched gp120 antigens in a subset of vaccinees from each region. Strikingly, the viral vector–elicited antibodies were significantly different from the bulk antibody glycans by PCA analysis (Fig 3A). Specifically, no overlap was observed in the overall glycan profiles of antigen-specific (maroon) and bulk antibodies (blue) (Fig 3A, left). Furthermore, all glycan types were found to be significantly different in vaccine-specific antibodies compared to bulk antibody glycosylation, with vaccine-specific glycans being significantly more agalactosylated, mono-galactosylated, sialylated and bisected but less fucosylated and di-galactosylated (Fig 3B). As illustrated in the loadings plot (Fig 3A, right), this separation was driven primarily by differences in fucosylation and sialylation. This analysis shows that antigen-specific antibody glycosylation is specific and driven by the immune signals delivered at the time of immunization.

### Vaccine-induced antibodies overcome regional bulk antibody glycosylation differences

To ultimately determine whether antibody glycosylation is programmed at an antigen-specific level in a reproducible manner independent of regional differences in bulk circulating Fc glycosylation, we next aimed to determine whether the regional differences observed in bulk Fc glycosylation were also present in antigen-specific antibody glycoprofiles. Remarkably, no differences among all three geographical locations were observed for any sugar in the antigen-specific antibody glycans (Fig 3C), strongly arguing that the viral vector-based vaccine induced a single glycan profile on vaccine-induced antibodies that is independent of pre-existing antibody glycan profiles. While a trend toward reduced addition of the b-GlcNAc was observed in the antigen-specific antibodies in the Kenyan/Rwandan vaccinees, these levels were opposite to those observed in the bulk antibody profiles (Fig 2A), arguing for a directed change away from the bulk antibody glycan profiles among the vaccine-induced antibodies. In addition, as expected, HA-specific antibody glycans were different across regions at each timepoint tested (cross-sectionally), but did not change due to vaccination (Fig 3D), indicating that vaccine-induced glycan changes are restricted to the vaccine antigen-specific antibody population, and does not globally affect the humoral immune response. Thus collectively, these data strongly argue that vaccine-induced glycosylation changes are highly restricted to the *de novo*-induced antigen-specific antibody response independent of baseline geographical regions in antibody glycosylation, suggesting that antigen-specific antibody glycosylation is induced at the time of B cell priming in a uniform and directed manner.

### Different immune signals during antigen exposure elicit distinct glycosylation profiles

**Fig 4:**
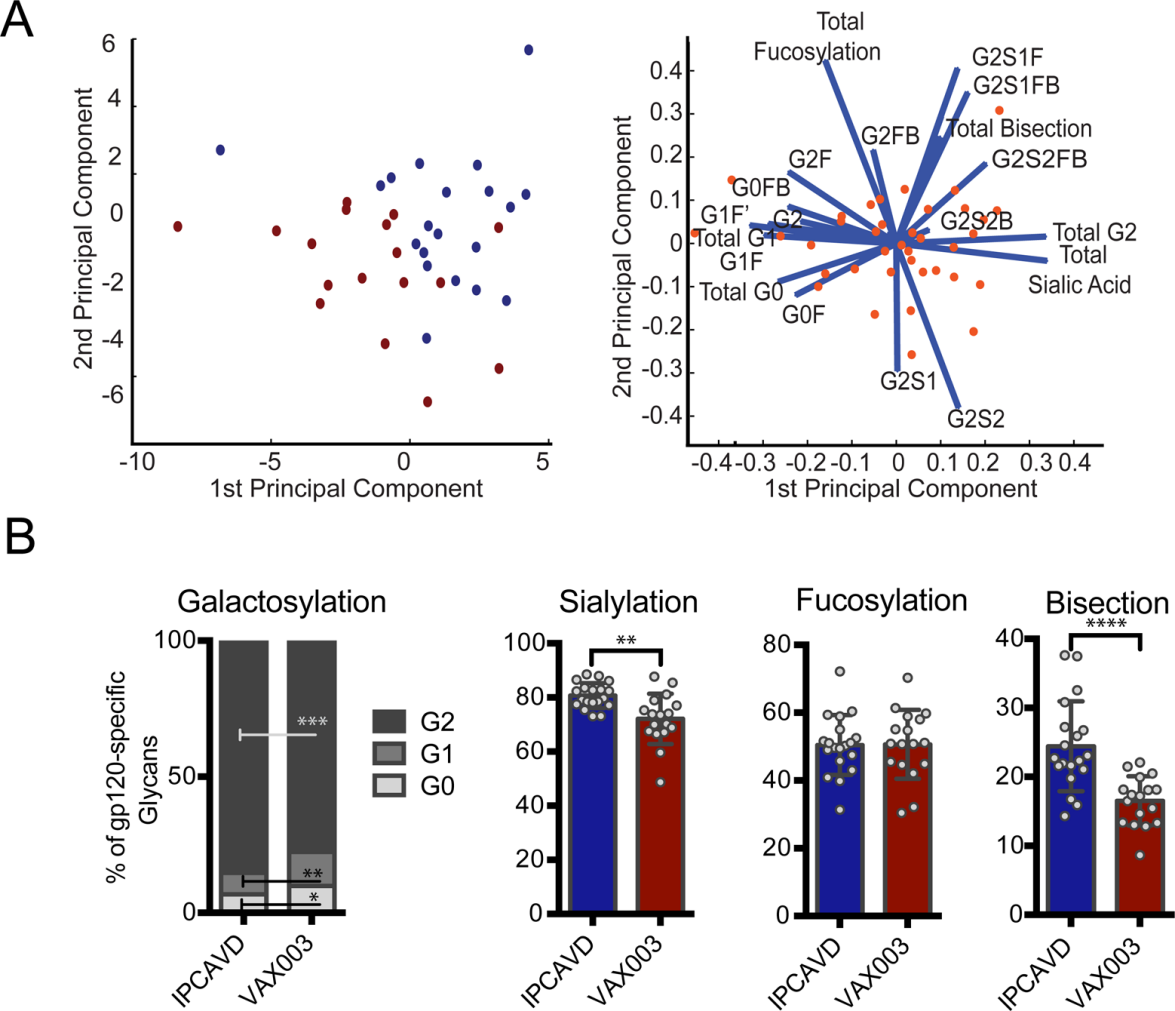
Different vaccines induce distinct vaccine-elicited antibody glycosylation profiles. (A) The antigen-specific IgG glycans from the B003/IPCAVD-004/HVTN 091 (blue) and VAX003 (maroon) studies were compared using PCA. This analysis described 56% of the variation. (B) The mean gp120-specific glycan profiles induced by the B003/IPCAVD-004/HVTN 091 (n = 19) and VAX003 (n = 17) trials were compared using the Mann-Whitney *U* test (*p<0.05, **p<0.01, ***p<0.001, ****p<0.0001).

Given that the adenovirus-vectored vaccines elicited similar glycosylation patterns on vaccine-specific antibodies in all tested vaccinees, we aimed to determine whether the observed antigen-specific glycan profiles are conserved across all *de novo*-induced antibodies or if they are tuned differentially by distinct immunogens at the time of vaccination. Antigen-specific glycan profiles of antibodies isolated from recipients of the VAX003 vaccine trial (an alum-adjuvanted recombinant gp120 vaccine that induced high titer vaccine-specific antibody responses) were compared with glycan profiles induced in the B003/IPCAVD-004/HVTN091 experimental vaccine trial. Interestingly, using PCA, we observed significant separation of the gp120-specific antibody glycosylation profiles across the two vaccines for galactose, sialic acid, and b-GlcNAc (Fig 4A). Specifically, the B003/IPCAVD-004/HVTN 091 vaccine trial induced more anti-inflammatory glycan structures, with higher proportions of di-galactosylated, and sialylated glycans compared to antibodies induced in the VAX003 trial (Fig 4B). The viral vector used in B003/IPCAVD-004/HVTN 091 also induced an increased proportion of bisected glycan structures, which are known to elicit greater ADCC activity and therefore increased functionality [13]. However, no differences were observed in fucose content across the two vaccine trials. Thus, the viral vector used in B003/IPCAVD-004/HVTN091 induced a less inflammatory but highly functional antibody glycan profile compared to the poorly functional but highly inflammatory antibody glycan profiles induced in the alum-adjuvanted VAX003 study.

All together, these data demonstrate that different immune signals delivered at the time of antigen exposure can specifically tune the glycosylation of antigen-specific antibodies in a signal-specific manner. Furthermore, the same antigen, HIV gp120, can induce differentially glycosylated antibodies in the presence of distinct immune signals. Given the importance of glycosylation in modulating antibody effector functions, these data suggest that inflammatory signals at the time of B cell priming can direct antibody glycosylation, aimed at tuning antibody effector function to target antigens in a pathogen-specific manner. Importantly, these data highlight that next-generation vaccine design strategies may selectively tune antibody effector function via modulation of antibody glycosylation.

## Discussion

Unlike subclass selection, which irreversibly changes the constant domain, antibody glycosylation represents a flexible and powerful mechanism by which the immune system naturally finely tunes antibody effector function. However, while significant changes in antibody glycosylation have been reported on bulk circulating antibodies in the setting of chronic inflammatory diseases [27] and on antigen-specific antibodies [15,32], it is still unclear whether the immune system naturally and selectively tunes antibody glycosylation in an antigen-specific manner. Here, we show differential glycosylation on distinct antigen- and pathogen-specific antibodies isolated from the same individuals (Fig 1), suggesting that the selection of antibody glycan profiles may be determined at the time of B cell priming as a means to specifically tune antibody effector activity to eliminate individual targets in an antigen/pathogen-appropriate manner. Moreover, we show that antibody glycosylation can be actively influenced via vaccination, overcoming different baseline circulating antibody glycome differences among vaccinees (Fig 2), to generate a specific antibody glycan profile within the vaccine-specific antibody subpopulation (Fig 3). Finally, distinct differences were observed in antibody glycan profiles among antibodies induced by different vaccines (vectored versus protein-only), highlighting the critical nature of distinct priming signals in directing the glycan profiles of antigen-specific antibodies (Fig 4). Given that antigen-specific antibodies represent only a small percent of the total circulating antibodies, which are composed of swarms of distinct epitope-specific antibodies, it is unlikely that antigen-specific antibody glycan shifts would influence the overall circulating glycome. However, differential antigen-specific antibody glycosylation clearly reflects differences in selective immune programming directed against distinct pathogens/antigens aimed at harnessing the broad Fc effector functional potential of the humoral immune response.

The differential selection of antigen-specific antibody glycosylation profiles may be a means by which the humoral immune system customizes and selectively arms antibodies with extra-neutralizing effector functions that are more effective in controlling and clearing particular pathogens. While the magnitude of the observed glycan changes appear small, they are highly significant, and even small shifts were clearly associated with robust changes in antibody effector functions (Fig 1C). Given that antibodies function as polyclonal swarms in immune-complexes, small glycan changes may have profound effects on innate immune Fc-effector cell functionality by simply skewing Fc-receptor/complement activation towards more desirable functions. Interestingly, some of the associations reflect previously identified relationships described for monoclonal therapeutic functional enhancement (low fucose = ADCC). However, additionally, novel glycan profile shifts that tracked with enhanced viral inhibition (G1), phagocytosis (G0), ADCC (SA), and complement activation (F and SA). Additionally, HA-specific antibodies exhibited more galactosylated, sialylated (less inflammatory), and afucosylated (more ADCC) glycan profiles (Fig 1A), suggestive of an antibody glycan profile tuned to promote rapid NK cell cytotoxicity via FCGR3A [12] in the absence of high levels of inflammation [33]. By contrast, gp120-specific antibodies exhibited a more inflammatory profile marked by low galactosylation and sialylation with higher levels of fucosylation and b-GlcNAc incorporation, the latter of which has been implicated in driving ADCC and complement activation [12,13]. Because the containment of influenza virus occurs predominantly within the lung [34], where rapid killing in the absence of excessive inflammation may be required to avoid immunopathology, an anti-inflammatory but ADCC-inducing antibody glycan would be highly protective. Conversely, non-neutralizing control of HIV may occur both in the blood as well as in lymphoid and gut tissues [35], where excessive pathology has been documented [36,37]. However, whether less inflammatory and functional antibodies targeting HIV could provide enhanced control of HIV is unclear.

Defining protective glycan structures against other pathogens that infect via mucosal tissues may point to potential antibody effector profiles that could provide enhanced early containment of HIV. Yet, differences were observed in antigen-specific antibody glycosylation, strongly suggesting that the immune response evolves a highly specific antibody glycoprofile to target each pathogen, engineering the correct glycans to generate antibodies that are the most functional for particular pathogen. Thus, since the choice of N-linked glycan on the antibody is both directable and crucial to determining the functionality of antibodies, a deeper understanding of how and why particular antibody glycans are built up is crucial for understanding mechanisms to actively manipulate and control the bioactivity of antibodies via vaccination. Moreover, as vaccine-specific antibody glycosylation profiles were similar within vaccinees, irrespective of their baseline bulk antibody glycosylation profile, suggests that B cell programming of antibody glycosylation must occur in an environment that is unaffected by baseline inflammatory differences that may be driven by different diets, microbiomes, or co-infections, [25,30,31]. Furthermore, alterations in antibody glycosylation during vaccination clearly occurs in an antigen-specific manner, as HA-specific antibody glycosylation profiles were unaltered, suggesting that antibody glycosylation must require some level of B cell receptor triggering. Yet, because distinct vaccinations elicited discrete antibody glycan profiles, it is likely that unique inflammatory signals, delivered via TLRs or cytokines, at the time of BCR engagement, must play a critical role in tuning the antibody glycoprofile towards particular effector function, potentially allowing B cells to integrate information and program function according to the microbial origin of the antigen to which they are selected.

Previous genome wide data have identified a small number of single nucleotide polymorphisms that track with altered systemic antibody glycosylation [38]. However, here we observed highly significant regional differences in the circulating antibody glycome, that extend far beyond the frequencies at which these SNPs occur within populations, suggesting strongly the critical importance of environmental influences on modulating and shaping circulating humoral immune profiles. Whether these antibody-glycome profiles are imprinted gestationally, related to nutritional differences or linked to differences in co-infections remains to be determined. Furthermore, whether this profile is reversible (upon relocation), and if it fluctuates over time also remains unclear. Given the critical nature of non-specific antibody glycosylation in therapeutically reducing inflammation, as is observed with IVIG [19,20], understanding the key modulators of bulk glycosylation may not only help tune immunologic inflammation but also provide insights into populations at risk for particular infection, malignancies, or autoimmune disease.

Irrespective of the baseline circulating antibody glycome differences, vaccination resulted in the rapid selection of a single vaccine-specific glycan profile, suggesting that vaccination can select for uniform protective humoral immune profiles globally. However, whether these antibody glycan profiles change over time and whether they are preserved and can be recalled is unclear. Because humoral immune responses are composed of different waves of plasmablasts, it is plausible that the peak vaccine-specific antibody glycan profile may represent a particular wave of glycan profiles that later matures over time, concurrent with the loss of specific plasmablast populations. Thus future longitudinal vaccine studies may provide enhanced insights into the specific antibody-glycoprofiles that may be seeded into the long-lived plasma cell pool, which are aimed at conferring immunologic memory until pathogen re-exposure. Moreover, understanding how antibody glyco-profiles may be tuned in memory-B cells that give rise to new waves of plasmablasts, through prime-boosting or via reaction to distinct adjuvants, offers a unique opportunity to fine tune antibody effector activity. Thus these data highlight the critical need to more fully understand the signals and mechanisms that program glycan profiles naturally and via vaccination to define the specific vaccines, adjuvants, and/or vectors that can selectively induce glycan perturbations linked to desired antibody effector functions.

This study is the first to clearly demonstrate that the immune system actively tunes antibody glycosylation in an antigen-specific manner via the delivery of specific signals at the time of antigen exposure, in a BCR-dependent manner, independent of baseline inflammatory differences. Whether host-genetics or baseline risk factors may impact B cell programming and thereby the ability to generate specific antibody glycan profiles is unknown. However given the emerging interest in modulating antibody effector activity against HIV, and other pathogens, novel strategies able to actively modulate antibody effector function in vivo are highly desirable. Thus the data presented here demonstrate that vaccination can harness antibody effector function via the regulation of antibody glycosylation offering a novel route to improve future vaccines that may provide protection from HIV and beyond.  

## Methods

### Vaccine and patient cohorts

HIV-positive patients were recruited through the Ragon Institute at Massachusetts General Hospital. A total of 197 HIV-positive subjects, balanced for sex and age, were included in this study, including 48 elite controllers (<75 copies RNA/ml) [39], 64 viremic controllers (75–2000 copies of RNA/ml), 44 HIV-positive patients on antiretroviral therapy (<70 copies RNA/ml), and 41 untreated HIV-positive patients (>70 copies RNA/ml). The B003/IPCAVD-004/HVTN 091 vaccine trial (clinicaltrials.gov ID: NCT01215149) was a safety and immunogenicity trial of a combination of adenovirus vectors (Ad26 and Ad35) expressing gp120 ENVA. Adenoviral vectors were administered at seven sites in three regions: the United States, East Africa, and South Africa. Low-risk, HIV-negative adults received two intramuscular doses of one or the other vector, and all samples used in this study were collected at peak immunogenicity, two weeks after final immunization (manuscript in preparation). The VAX003 trial was a phase III efficacy trial administered in Thailand in a high-risk population of intravenous drug users (clinicaltrials.gov ID NCT00006327). This trial used seven doses of AIDSVAX B/E, a recombinant gp120 clade B/E, with alum as the adjuvant. All samples used in this study were collected at peak immunogenicity, two weeks after the final vaccination.

### Ethics statement

The study was reviewed and approved by the Massachusetts General Hospital Institutional Review Board, and each subject gave written informed consent. All samples were collected and used with approval from local the Institutional Review Boards and appropriate national regulatory authorities [18]. The HIV positive patient cohorts were approved by the Massachusetts General Hospital institutional review board. The ICPAVD 004 trial was approved by the Harvard Medical School institutional review board and each subject gave written informed consent. The VAX003 study was approved by the Bangkok Metropolitan Administration institutional review board and each subject gave written informed consent.

### Fc glycan preparation

Plasma was collected by the vaccine trial staff and stored at -80°C until use. IgG was isolated using Melon Gel IgG purification resin (Thermo Fisher) according to the manufacturer's instructions. Fc glycans were released and analyzed as described [40]. Briefly, whole antibodies were treated with IdeS protease (Genovis) to separate the Fab from the Fc and Fc glycans were released by PNGaseF and then dried down prior to fluorescent labeling with 8-aminopyrene-1,3,6-trisulfonate. Labeled glycans were run on a capillary electrophoresis machine and glycan peaks were assigned using labeled standards. A representative glycan spectra is depicted in S1 Fig and a table of peak assignments and names are listed in S1 Table.

### Antigen-specific antibody purification

Isolated IgG was passed over gp120 embedded columns (YU2 for HIV-positive subjects, EnvA for IPCAVD vaccinees, and a 1:1 mix of A244 and MN for VAX003 vaccinees; Immune Technology Corp.), p24 (HXBc2; Immune Technology Corp.) or HA (mix of HAΔTM H1N1 A/Solomon Islands/3/2006, HAΔTM H3N2 A/Wisconsin/67/x161/2005, HAΔTM H1N1 A/Brisbane/59/2007, HAΔTM H3N2 A/Brisbane/10/2007, HA1 H1N1 A/New Caledonia/20/99, HA B/Malaysia/2506/2004/0054P and HAΔTM B/Florida/2006; Immune Technology Corp.). The bound antibody was eluted from the column in 0.1 M citric acid, pH 2.9. The purified IgGs were treated with PNGaseF to release the attached N-linked glycans for analysis. To compare these antibodies to the bulk fraction, a subset of bulk IgG was processed without Fc separation as described above.

### Functional assays

Functional profiling was performed as described previous [41] Briefly, bulk IgG from chronic untreated HIV patients was purified using a Melon Gel IgG purification Kit (Thermo Scientific). Complement activation was measured via the recruitment of C3d to CEM-NKR target cells by flow cytometry following antibody labeling of gp120-adsorbed target cells. ADCC was quantified following the elimination of fluorescently labeled antibody-coated-gp120-adsorbed CEM-NKR cells by purified primary NK cells [8]. NK cell activation was assessed following the addition of purified NK cells to antibody adsorbed gp120-coated 96-well plates by flow-cytometry as the frequency of NK cells degranulating (CD107a upregulation), chemokine (MIP-1β) or cytokine (IFN-γ) secreting cells [8]. For ADCP was measured as the level of antibody-induced-gp120-coated fluorescent bead uptake by flow cytometry by THP-1 cells [42]. Finally, ADCVI was quantified as the difference in HIV-JRCSF replication in the presence or absence of antibodies in activated CD4 T cells in the presence of autologous primary NK cells and antibodies over 7 days [43].

### Statistics

Univariate data were analyzed using GraphPad Prism Version 6.0e for Mac (GraphPad Software, San Diego, California) for statistical significance and graphical representation. The statistical tests used are indicated for each figure. Heat maps were constructed using GENE-E (Brode Institute, Cambridge, MA). Multivariate analyses were performed using MATLAB and Statistics Toolbox Release 2013b (the MathWorks Inc., Natick, Massachusetts) and JMP Pro 11.00 (SAS, Cary, North Carolina).

## Supporting Information

S1 Fig Example capillary electrophoresis spectrum. Peaks identified as described in S1 Table.
(TIF)

S2 Fig Correlations between glycan types. Correlations between gp120- and HA-specific antibodies were determined for agalactosylation, mono-galactosylation, di-galactosylation, fucosylation, bisection and sialylation by linear regression. P values and R^2^ values are displayed on the graph. No correlations were significant.
(TIF)

S3 Fig HA-specific antibody glycosylation in HIV patients. Differences in glycosylation for HA-specific antibodies from chronic treated (orange), chronic untreated (pink), viremic controller (purple) and elite controller (green) HIV patients were compared using two-way ANOVA with Tukey’s multiple comparison test. No differences were significant.
(TIF)

S1 Table Glycan peak table. Structure, glycan name and CE peak assignment. GlcNAc = blue square, mannose = green circle, galactose = yellow circle, sialic acid = pink diamond, fucose = red triangle. Structures assigned to peaks in S1. ND = not detected.
(TIF)
