# Correction: Antigen-Specific Antibody Glycosylation Is Regulated via Vaccination

**DOI:** 10.1371/journal.ppat.1005694

**Published:** 2016-06-02

**Authors:** Alison E. Mahan, Madeleine F. Jennewein, Todd Suscovich, Kendall Dionne, Jacquelynne Tedesco, Amy W. Chung, Hendrik Streeck, Maria Pau, Hanneke Schuitemaker, Don Francis, Patricia Fast, Dagna Laufer, Bruce D. Walker, Lindsey Baden, Dan H. Barouch, Galit Alter

The authors would like to correct Figs 1, 2 and 3 as errors were introduced in the preparation of these figures. In Fig 1A, the labels for G0 and G2 in the Galactosylation key are switched in the left-hand side panel and the titles Bisection and Fucosylation are switched. The corrected Fig 1 is presented here. In Fig 2A, the titles Bisection and Fucosylation are switched. The corrected Fig 2 is presented here. In Fig 3B, the labels for G0 and G2 in the Galactosylation key are switched, the titles Bisection and Fucosylation are switched, and the bars for gp120 and Bulk in the Galactosylation graph are switched. The corrected Fig 3 is presented here. The authors confirm that these changes do not alter their findings.

**Fig 1:**
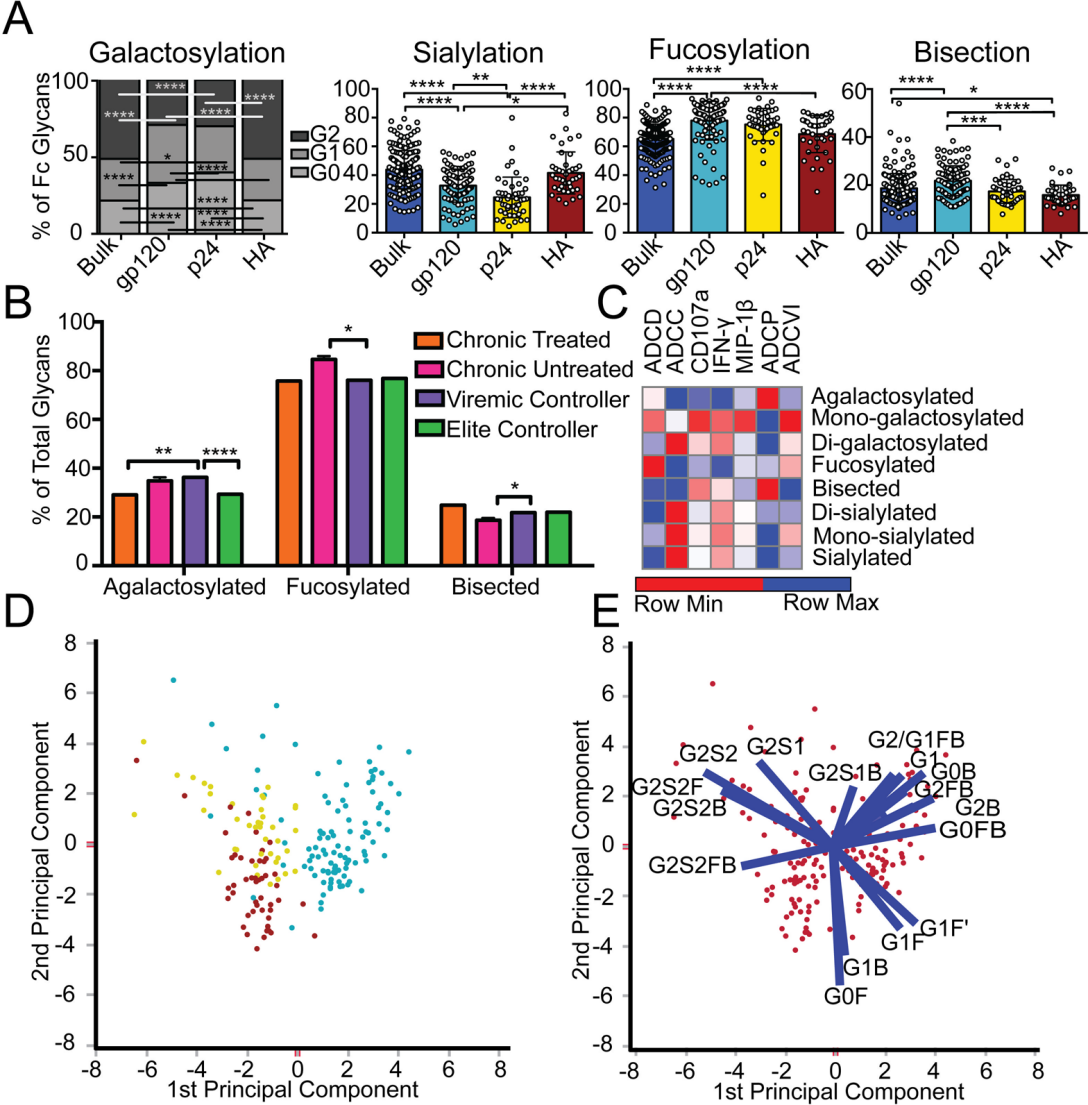
Antibody glycosylation is programmed in an antigen-specific manner. (A) Glycosylation was assessed on whole bulk-circulating and antigen-specific IgG antibodies directed against gp120 (n = 103), p24 (n = 47), and HA (n = 40) isolated from a cohort of 193 HIV-infected subjects. The dot plots represent the percent of glycan structures that contain galactose (G0 = agalactosylated, G1 = mono-galactosylated, G2 = di-galactosylated) or that contain sialic acid (S = sialylated), fucose (F = fucosylated), or a bisecting GlcNAc (B = bisected). Differences between groups were compared using Kruskal-Wallis test with Dunn’s multiple comparison’s test (*p<0.05, **p<0.01, ***p<0.001, ****p<0.0001). (B) Differences in glycosylation for gp120-specific antibodies from chronic treated (orange), chronic untreated (pink), viremic controller (purple) and elite controller (green) HIV patients were compared using two-way ANOVA with Tukey’s multiple comparison test (*p<0.05, **p<0.01, ***p<0.001, ****p<0.0001). (C) Antibody glycosylation levels on gp120-specific antibodies among chronic HIV-infected individuals were measured along with seven different gp120-specific effector functions. The correlations between gp120-specific antibody glycosylation and gp120-specific functional parameters were assessed using spearman correlations. Antibody dependent complement deposition (ADCD), antibody dependent cellular cytotoxicity (ADCC), antibody mediated NK cell activation (degranulation-CD107a, IFN-γ, or MIP-β secretion), antibody dependent cellular phagocytosis (ADCP), and antibody dependent cellular viral inhibition (ADCVI) are depicted. Significant correlations were identified for: Fucose—ADCC*, Di-sialylated—ADCC**, mono-galactosylated—ADCP*, Di-galactosylated—ADCP**, Bisection—ADCP* and Mono-sialylated—ADCP** (*p<0.05, **p<0.01, ***p<0.001, ****p<0.0001). (D), (E) To gain a multivariate sense of the overall glycan profile differences among antigen-specific antibodies, antigen-specific antibody glycan profiles (gp120- (teal), HA- (yellow) and p24- (red) specific antibodies) were compared using principle components analysis (PCA). Each dot on the score plot (D) represents an antigen-specific antibody glycan profile from a single individual and the loadings plot (E) shows the contribution of individual analyzed glycan structures to driving the separation between the antigen-specific antibody glycan profiles. Vector length represents the magnitude of individual glycan structure effects on overall separation in antibody glycan profiles, with longer vectors represent features that are further from the mean and that drive a larger effect on separating antibody glycan profiles. Moreover, location on the loadings plot is identical to location on the score plot, determined by the collective influence of all vectors. This analysis accounts for 42.9% of the glycosylation variation across the antigen-specific antibody specificities.

**Fig 2:**
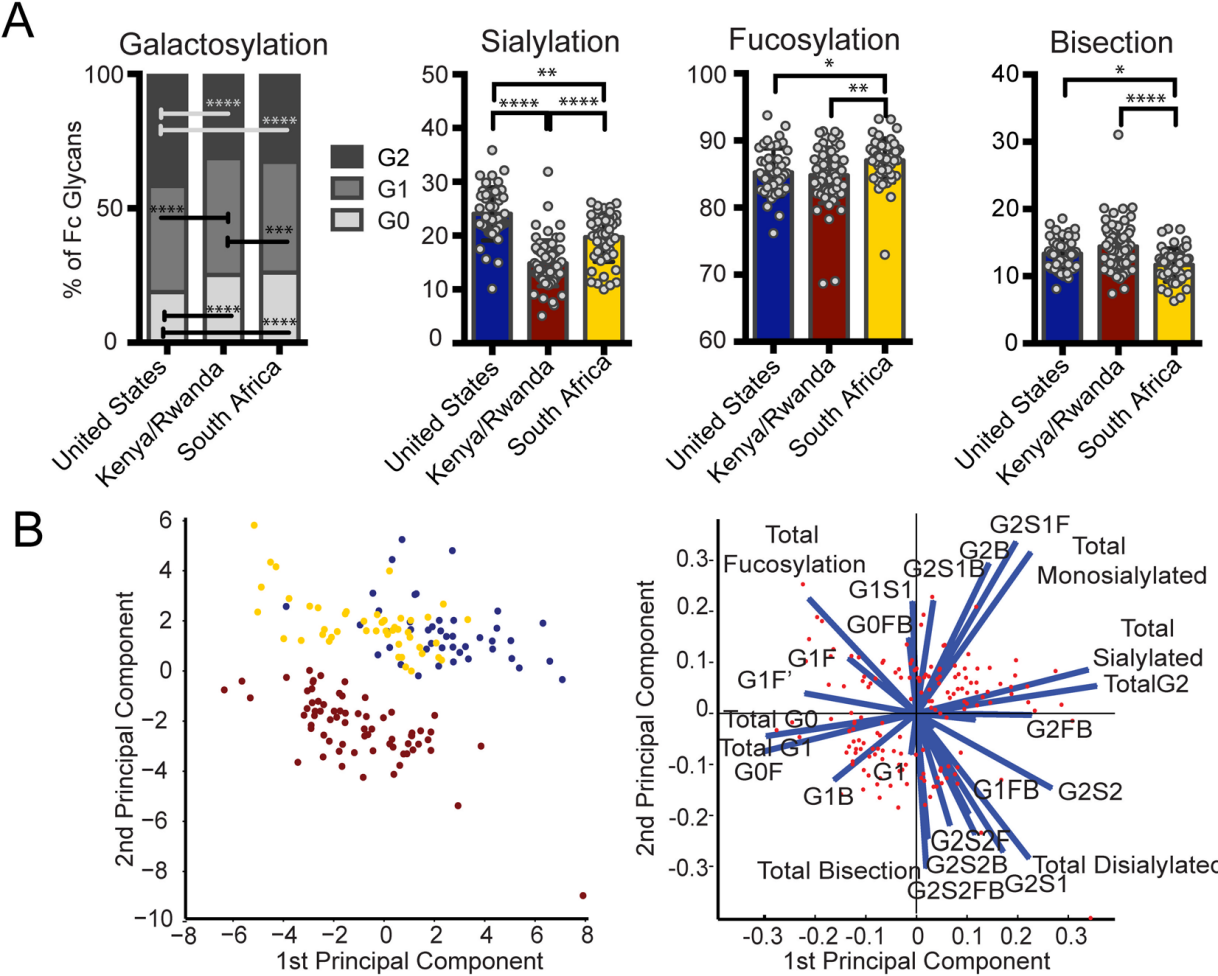
Geographic location affects bulk IgG glycosylation. Bulk IgG glycosylation was assessed in subjects from three regions: Unite States (blue, n = 43), Kenya and Rwanda (maroon, n = 69), and South Africa (yellow, n = 47). (A) Bulk antibody Fc glycosylation in vaccine recipients from each of the three regions was measured via capillary electrophoresis, and the mean proportion of total galactosylated, sialylated, fucosylated, and bisected structures was compared using Kruskal-Wallis one-way ANOVA (*p<0.05, **p<0.01, ***p<0.001, ****p<0.0001). (B) Multivariate comparison of antibody Fc glycosylation among the three geographic sites was performed using PCA. The score plot (left panel) depicts the principal component analysis of samples collected in the three regions (each dot represents a vaccinee, and colors are as described above), and the loadings plot of the PCA (right panel) shows the contribution of particular glycan structures to driving the observed separation, where longer arrows signify a greater contribution to separating glycan profiles. This PCA describes 55% of the total variance among these samples.

**Fig 3:**
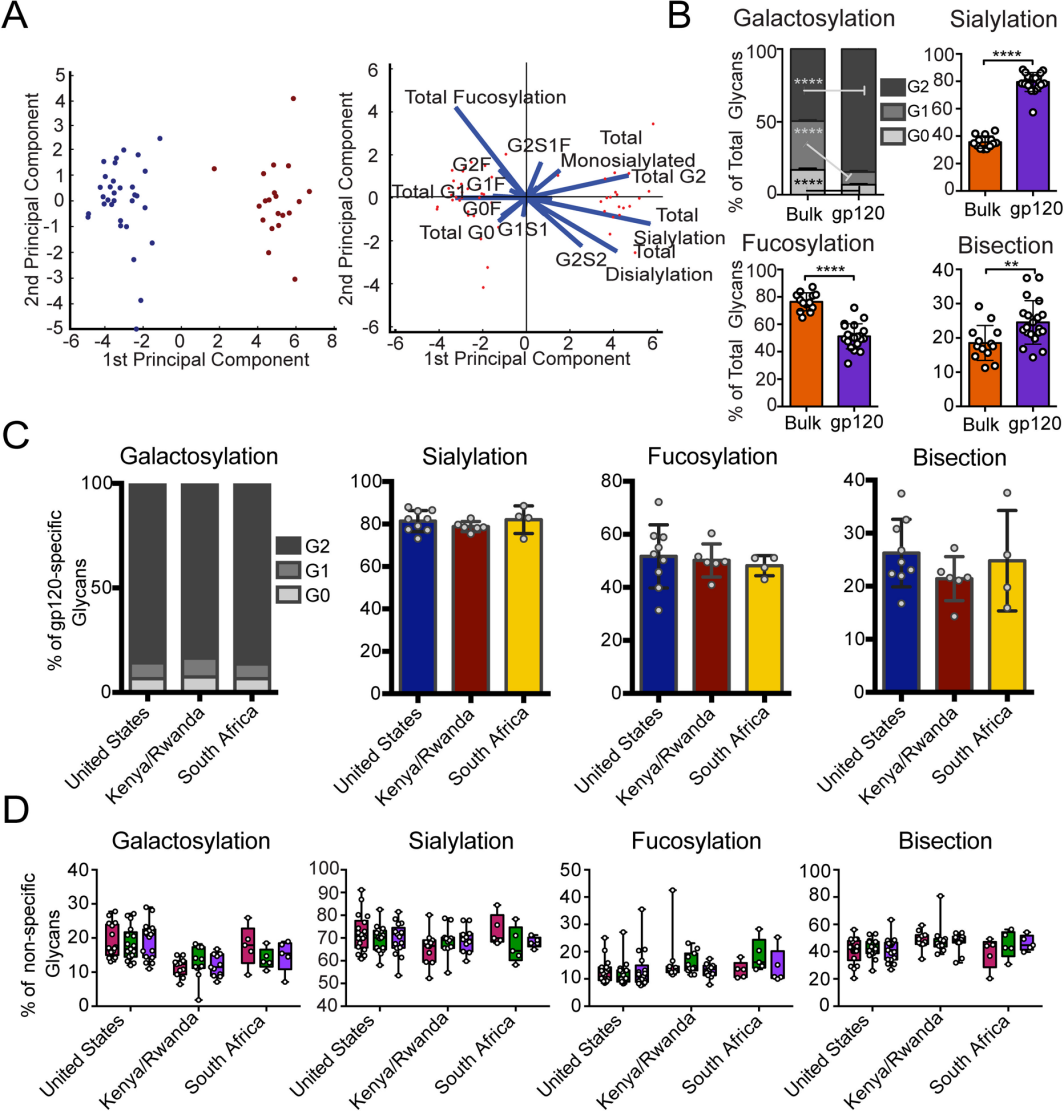
Vaccine-elicited antibody glycosylation profiles are distinct from bulk antibody glycosylation. Viral vector–induced gp120-specific and influenza specific antibodies were isolated from vaccinees, and the attached glycans were analyzed by capillary electrophoresis. (A) Multivariate PCA was used to compare bulk antibody glycoprofiles (blue, n = 32) and vaccine-elicited antigen-specific antibody glycoprofiles (maroon, n = 20), and both the scores plot (left) and loadings plot (right) are shown. This analysis describes 69% of the variation. (B) The mean proportions of bulk and vaccine-elicited antibody glycan were compared using students two-tailed paired t tests (n = 13 for bulk, n = 20 for gp120 (*p<0.05, **p<0.01, ***p<0.001, ****p<0.0001) (C) The mean proportions of vaccine-elicited antibody glycan structures were compared across vaccine groups using Kruskal-Wallis ANOVA (n = 9 for United States, n = 6 for Kenya/Rwanda, n = 4 for South Africa). No statistically significant differences were found. (D) The mean proportions of influenza-specific antibody glycans at baseline (magenta), post-first (green), and post-boost (purple) vaccine timepoints were compared using non-parametric two-way ANOVA (n = 18 for United States, n = 11 for Kenya/Rwanda, n = 5 for South Africa). No significant differences were found between the three timepoints for either antigen or glycan type.
