# Supplementary figures and images for: Antigen-Specific Antibody Glycosylation Is Regulated via Vaccination
Source: PLoS Pathog. 2016 Mar 16;12(3):e1005456. doi: 10.1371/journal.ppat.1005456 (PMC4794126; doi:10.1371/journal.ppat.1005456)

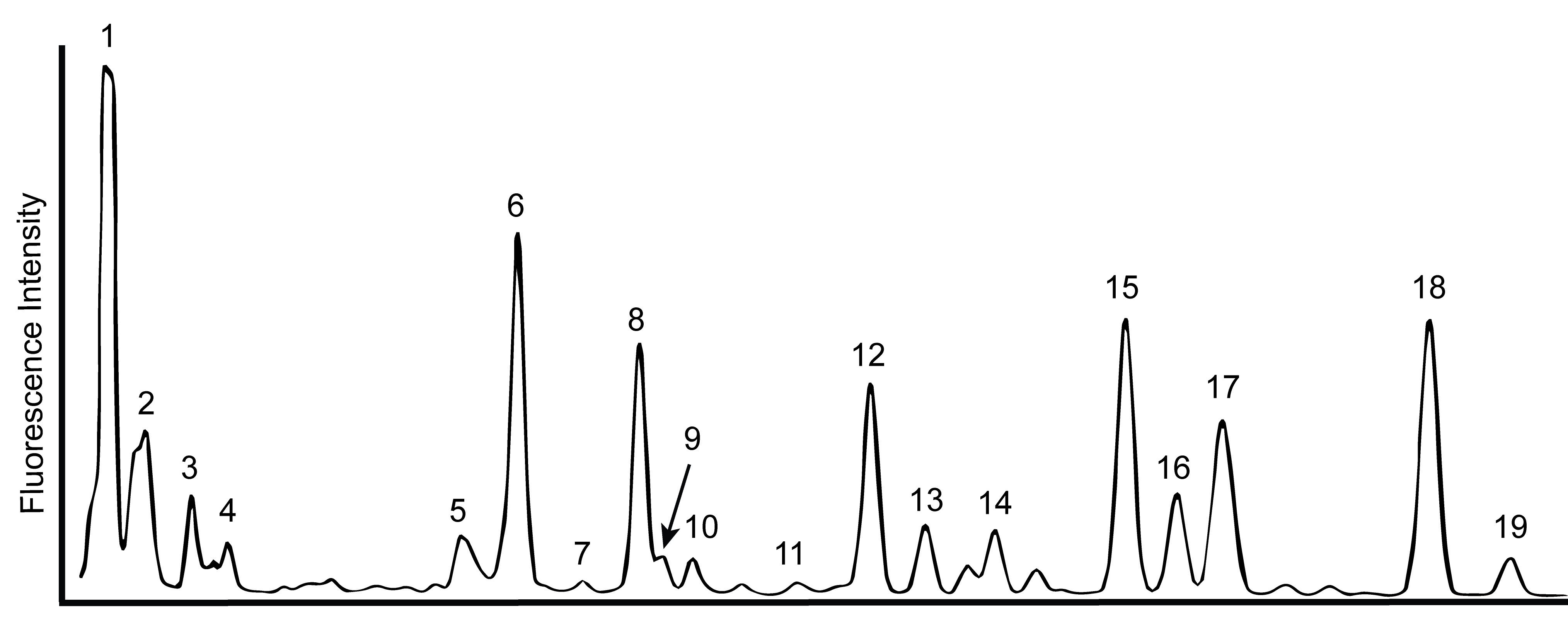

Supplement: S1 Fig — Peaks identified as described in S1 Table. (TIF) [file ppat.1005456.s001.tif]

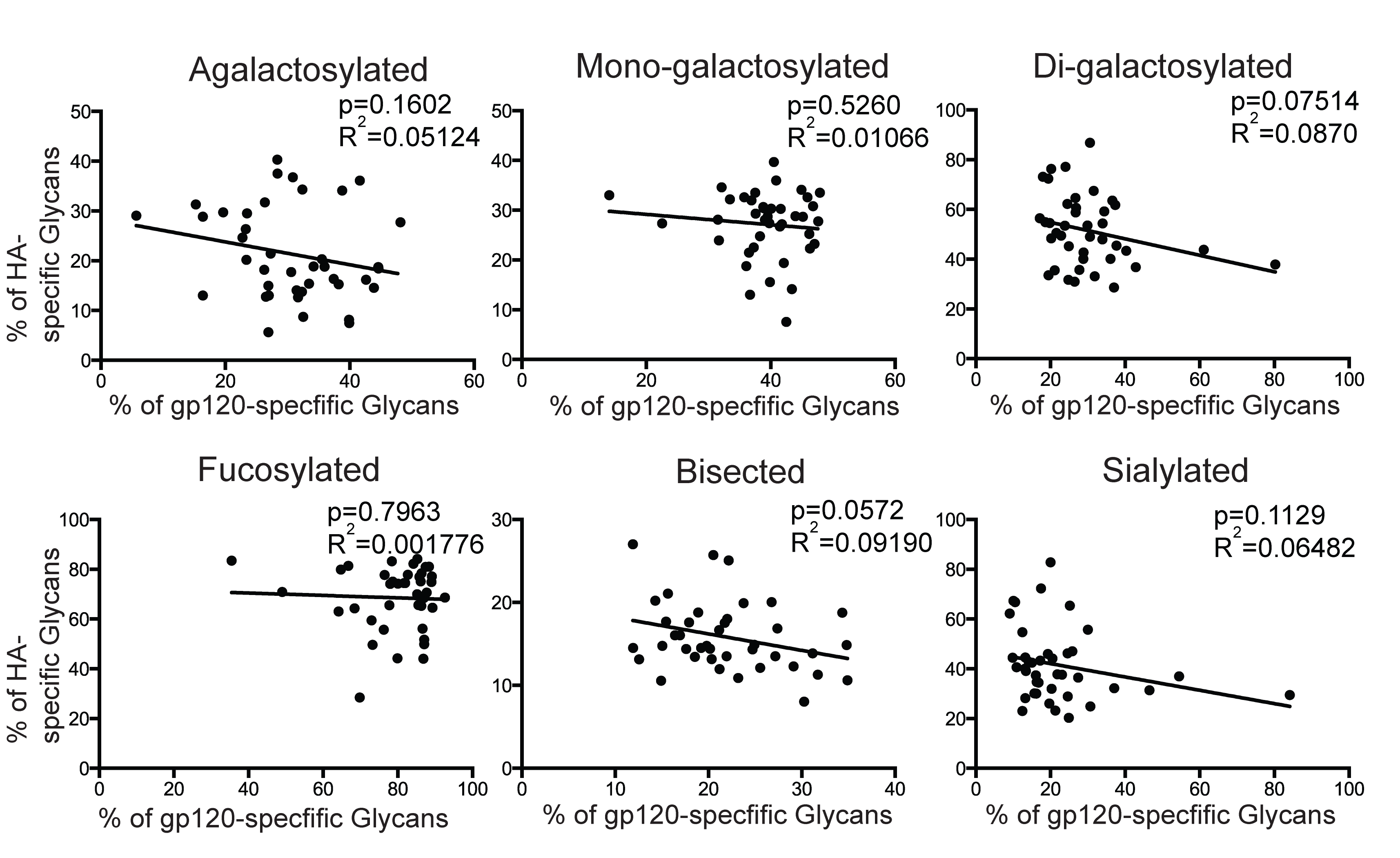

Supplement: S2 Fig — Correlations between gp120- and HA-specific antibodies were determined for agalactosylation, mono-galactosylation, di-galactosylation, fucosylation, bisection and sialylation by linear regression. P values and R2 values are displayed on the graph. No correlations were significant. (TIF) [file ppat.1005456.s002.tif]

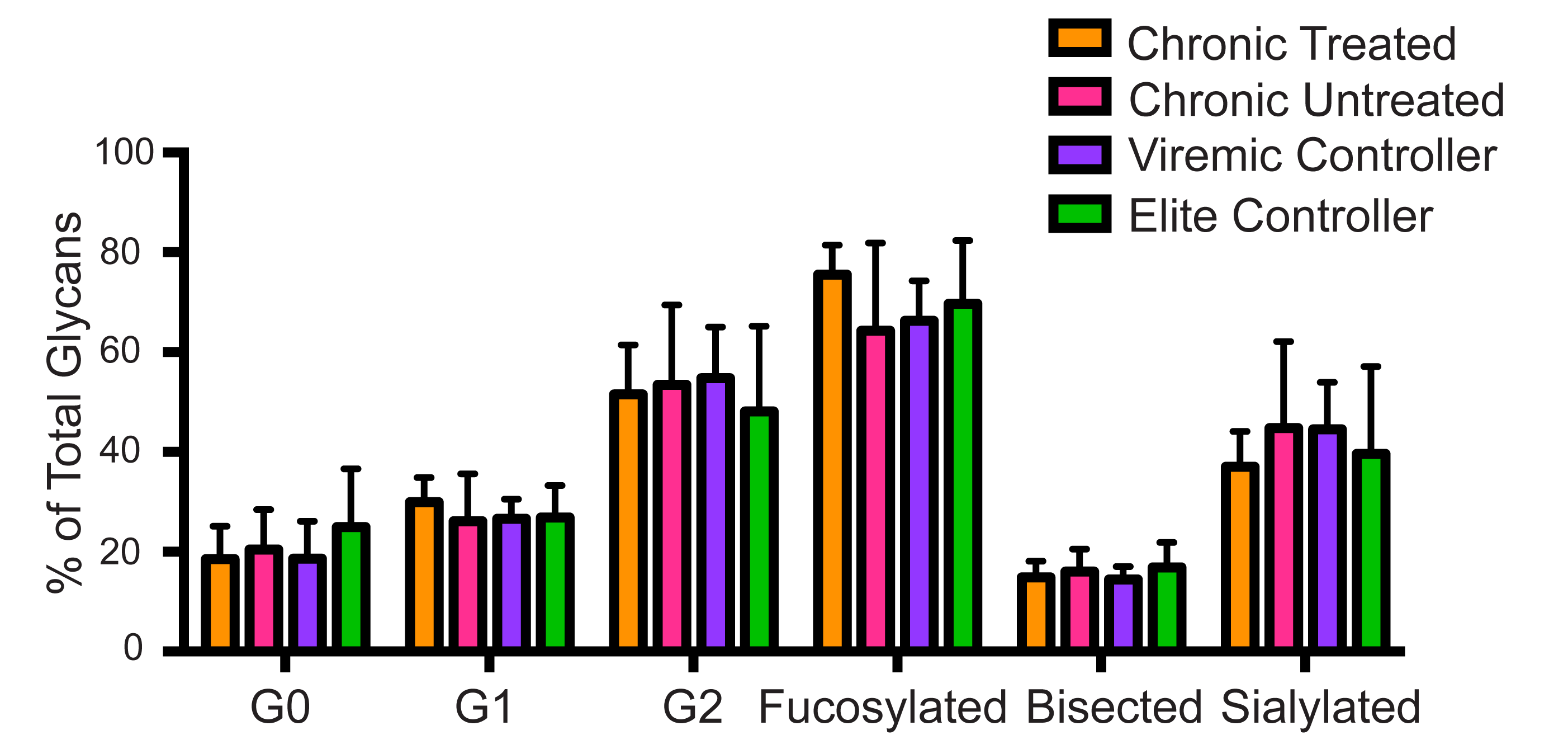

Supplement: S3 Fig — Differences in glycosylation for HA-specific antibodies from chronic treated (orange), chronic untreated (pink), viremic controller (purple) and elite controller (green) HIV patients were compared using two-way ANOVA with Tukey’s multiple comparison test. No differences were significant. (TIF) [file ppat.1005456.s003.tif]

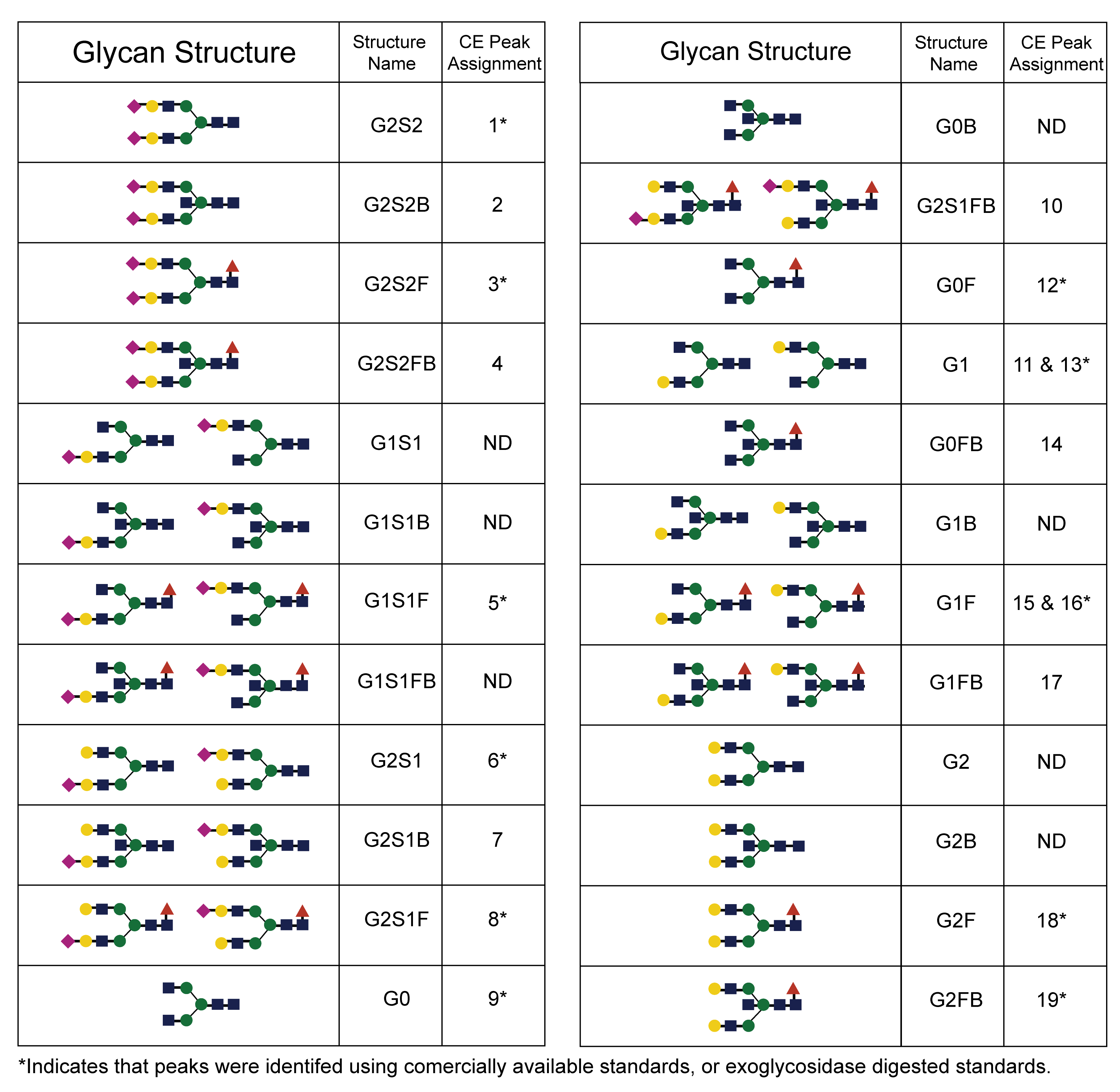

Supplement: S1 Table — Structure, glycan name and CE peak assignment. GlcNAc = blue square, mannose = green circle, galactose = yellow circle, sialic acid = pink diamond, fucose = red triangle. Structures assigned to peaks in S1. ND = not detected. (TIF) [file ppat.1005456.s004.tif]
